# “I Just Want You to Hear That Term”: Characterizing Language Used in Fetal Cardiology Consultations

**DOI:** 10.3390/jcdd10090394

**Published:** 2023-09-13

**Authors:** Kelsey Schweiberger, Kelly W. Harris, Ann Kavanaugh-McHugh, Abdesalam Soudi, Robert M. Arnold, Jessica S. Merlin, Nadine A. Kasparian, Judy C. Chang

**Affiliations:** 1 Department of Pediatrics, University of Pittsburgh School of Medicine, Pittsburgh, PA 15213, USA; harriskw@upmc.edu; 2 Department of Pediatrics, Vanderbilt University Medical Center, Nashville, TN 37232, USA; ann.kavanaugh-mchugh@vumc.org; 3 Department of Linguistics, University of Pittsburgh, Pittsburgh, PA 15260, USA; soudia@pitt.edu; 4 Section of Palliative Care and Medical Ethics, Department of General Internal Medicine, Palliative Research Center, University of Pittsburgh School of Medicine, Pittsburgh, PA 15213, USA; arnoldrm@upmc.edu (R.M.A.); merlinjs@upmc.edu (J.S.M.); 5 The Heart Institute, Cincinnati Children’s Hospital, Cincinnati, OH 45229, USA; nadine.kasparian@cchmc.org; 6 Department of Pediatrics, College of Medine, University of Cincinnati, Cincinnati, OH 45267, USA; 7 Division of Behavioral Medicine & Clinical Psychology, Cincinnati Children’s Hospital, Cincinnati, OH 45229, USA; 8 Department of Obstetrics, Gynecology, and Reproductive Sciences, University of Pittsburgh School of Medicine, Pittsburgh, PA 15213, USA; chanjc@upmc.edu

**Keywords:** prenatal diagnosis, congenital heart disease, communication

## Abstract

The way clinicians communicate with parents during pregnancy about congenital heart disease (CHD) can significantly influence parental understanding of and psychological response to the diagnosis. A necessary first step to improving communication used in fetal cardiology consultations is to understand and describe the language currently used, which this paper aims to do. Nineteen initial fetal cardiology consultations with parents were audio-recorded, transcribed verbatim, and coded by two independent coders. A codebook was inductively developed and applied to all transcripts. The finalized coding was used to characterize fetal cardiologists’ language. We identified four discourse styles employed in fetal cardiology consultations: small talk, medical, plain, and person-centered. Plain language was used to define and emphasize the meaning of medical language. Person-centered language was used to emphasize the baby as a whole person. Each consultation included all four discourse styles, with plain and medical used most frequently. Person-centered was used less frequently and mostly occurred near the end of the encounters; whether this is the ideal balance of discourse styles is unknown. Clinicians also used person-centered language (as opposed to disease-centered language), which is recommended by medical societies. Future studies should investigate the ideal balance of discourse styles and the effects of clinician discourse styles on family outcomes, including parents’ decision-making, psychological adjustment, and quality of life.

## 1. Introduction

The way in which clinicians communicate with parents during pregnancy about complex congenital heart disease (CHD) can significantly influence parental understanding of and psychological responses to the diagnosis [1]. Fetal cardiology consultations provide an important opportunity to optimize this communication. One essential element of medical communication is the language used [2]. Efforts have been directed toward standardizing the medical language used to describe CHD to create greater consistency in the language used by health professionals and to allow for analysis of outcomes across institutions (e.g., diagnostic language and surgical outcomes) [3]. While standardized medical language is necessary for communication among clinicians, it is not always patient-friendly. The American Medical Association [4] and Agency for Healthcare Research and Quality [5] have created toolkits aimed at improving health communication that emphasize limiting the use of complex medical terms, utilizing person-first language, and avoiding victimizing, disease-first descriptions. Person-first language acknowledges the person first, then their diagnosis, such as “a child with congenital heart disease” rather than disease-first language, which acknowledges the disease first, followed by the person, such as “a congenital heart disease patient”. 

Beyond using person-first language, using plain language, defined as clear communication that the public can understand and use [6], has also been recommended to improve healthcare communication. However, despite these recommendations, the use of medical jargon is common in clinical encounters and is infrequently accompanied by an easily understandable explanation [7]. In contrast, clinicians who use non-medical terms are often given higher patient satisfaction ratings on patient communication [8], and the use of simpler language results in more effective patient-clinician communication, which can have positive downstream effects on health outcomes [9].

Within the field of fetal cardiology, our prior research described how parents thought hearing the term “heart difference” in lieu of “heart defect” positively impacted their perspective when receiving a prenatal diagnosis of complex CHD [10]. Aside from this work, however, little is known about the language used during fetal cardiology consultations. Before developing any interventions to optimize the communication used when delivering these challenging diagnoses, we need to first understand and describe the language and communication currently used in these visits.

## 2. Materials and Methods

### 2.1. Design and Setting 

We conducted a qualitative study of audio recordings of initial fetal cardiology consultations. Participants were all people present during the visit, including clinicians, pregnant persons (who all self-identified as mothers, so hereafter referred to as mothers), and any partners or other support persons (hereafter referred to as partners). For any visits conducted using an interpreter, the transcription was completed using real-time English interpretation. Demographic data were collected by study personnel during the visit.

### 2.2. Participant Recruitment and Data Collection 

Clinicians were recruited from one tertiary care facility. All fetal cardiology clinicians who performed prenatal consultation visits for CHD were eligible and were invited to participate in the study via email, with the study details subsequently reviewed in person with each interested clinician. Eligible mothers were those who attended their first fetal cardiology appointment and had a referral diagnosis that included suspected CHD. All eligible participants were approached immediately prior to their scheduled clinic visit. All participants (mothers, partners, and clinicians) gave written informed consent and verbal permission for audio recording.

Initial fetal cardiology consultation visits included a fetal echocardiogram followed by counseling with a fetal cardiology clinician between May 2019 and August 2019. Audio recordings were completed using a digital voice recorder placed in the counseling room and initiated at the start of counselling after the completion of the fetal echocardiogram. This study was approved by the institutional review boards at the institutions participating in data collection and analysis.

### 2.3. Data Analysis 

Audio recordings of initial fetal cardiology consultations were professionally transcribed verbatim, and all personal identifiers were removed. The deidentified transcripts were analyzed by two coders (KS and KWH), and a preliminary codebook was created inductively [11]. The same two coders continued to code each transcript, refining the codebook systematically and iteratively with mentorship by an expert qualitative researcher (JCC). When thematic saturation was achieved (i.e., no new codes or changes to the codebook were noted), the finalized codebook was then applied to all transcripts by both coders. Any coding differences were adjudicated in full agreement. NVivo12 (QSR International; Burlington, MA, USA) was used to store and organize qualitative data.

Prior analyses have focused on conversation structure and the ways in which illness uncertainty was discussed [12,13]. In this analysis, we focused on the codes and sub-codes relating to the language used during initial fetal cardiology consultations. Our codebook included inductive codes related to the ways in which congenital heart disease was referenced and the use of anatomical and medical language throughout the consultations. We then used discourse analysis to identify categories of language use based on our findings.

In a post hoc analysis to identify the order of discourse styles used in each consultation, each transcript was coded by segment as one of the four discourse styles identified in the analysis by two coders (KS and KWH). Any coding differences were adjudicated in full agreement. Heat maps were created for each consult using coded segmented text to depict the order of discourse styles (Supplementary Figure S1). Trends across visits were further consolidated into a representative figure.

## 3. Results

**Figure 1:**
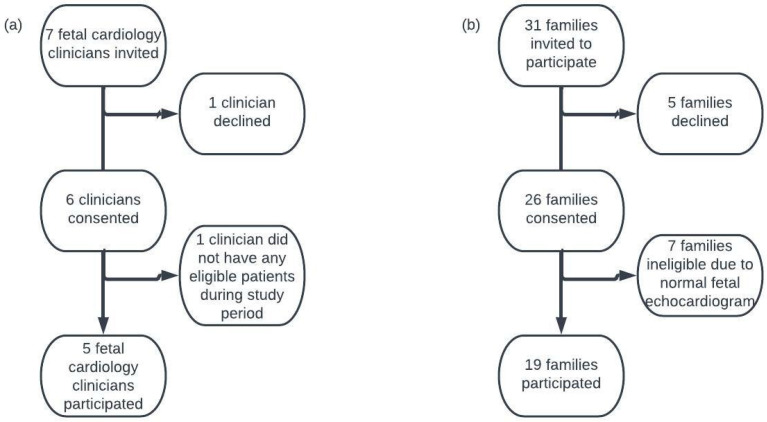
Recruitment flow diagram. (**a**) Fetal cardiology clinician recruitment diagram. (**b**) Family recruitment diagram.

Of the 7 fetal cardiology clinicians invited to participate in the study, 1 declined and 1 had no eligible patients during the study period. Five clinicians (4 cardiologists and 1 nurse practitioner) participated in the study. Of the 31 families invited to participate in the study, 5 declined participation and 7 families were not eligible based on fetal echocardiogram results (Figure 1); thus, we obtained 19 audio-recorded consultations. The 5 clinicians trained at 5 institutions across residency and fellowship and practiced at the current institution for a median of 9 years (interquartile range 16.5; Table 1). Most participating mothers self-identified as white and non-Hispanic (74%), identified English as their preferred language (79%), and had a partner accompany them (74%). Five (26%) self-reported a family history of CHD. Across the 19 consultations, 13 different cardiac diagnoses were discussed, and the counseling sessions had a median duration of 37 min (IQR: 25 min). Each consultation consisted primarily of the clinicians speaking; on average, 89% of the words were spoken. The following sections include illustrative quotations identified by clinician ID number (C1 to C5) and family ID number (F1 to F19).

**Figure 2:**
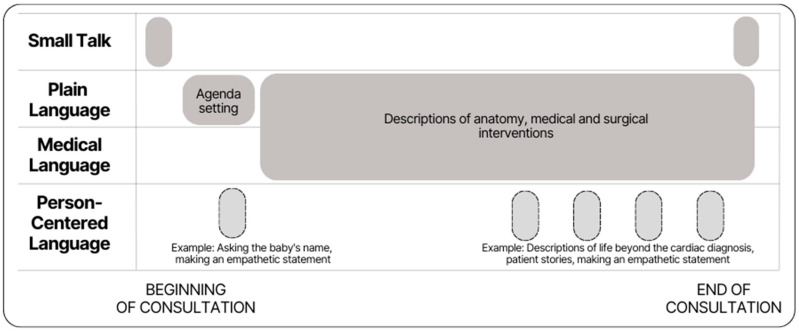
General sequence of discourse styles used throughout an initial fetal cardiology consultation. Use of discourse style is indicated by the gray boxes moving from beginning to the end of the consultation along the x-axis. Discourse style used intermittently are indicated with a hashed border.

The discourse styles used by fetal cardiology clinicians consisted of small talk, medical, plain, and person-centered language (Table 2). Medical language includes medical terminology or jargon. Plain language included clear, straight-forward communication using everyday words [6]. Person-centered language emphasizes the person prior to the disease (i.e., person-first language) or acknowledges the person’s experience as opposed to talking about them objectively. Small talk included polite conversation about non-sensitive subjects (e.g., the weather). While each consultation included all four discourse styles, plain language was used most frequently in all visits. Most clinicians began consultations with small talk, then set an agenda and introduced concepts using plain language, and then described anatomy and potential interventions using medical language accompanied by plain language explanations. Occasionally, clinicians used person-centered language at the beginning of the consultation, but mostly, they interspersed this language near the end of the consultation (Figure 2).

### 3.1. Use of Plain Language to Define and Explain Medical Terms

Plain language was used most frequently, often accompanying medical language the first time it was used (Figure 2). Plain language often functions to define and explain medical language for families. For example, one clinician said, “There’s a muscular ridge in between the pump and the way to the lungs and that is called pulmonary atresia and ‘atresia’ means ‘absent” (C1, F13). Similarly, descriptions of medical and surgical interventions included a large concentration of medical language accompanied by plain language descriptions such as, “We can do something called a catheterization where we pass long slender tubes about the size of spaghetti noodles, angel hair, through the blood vessels and into that ductus” (C1, F13). Plain language was also used to emphasize the meaning behind medical language. For example, “She has … a dysplastic valve, meaning the valve is just a little bit different … it’s … kind of narrow … making it … difficult for the blood to go forward” (C5, F4).

### 3.2. Use of Plain Language as a Substitute for Medical Language

Medical language regarding specific diagnoses was often introduced in the context of encouraging the family to become familiar with the term. For example, one clinician said, “So that valve does not close perfectly when the heart squeezes and that’s what an AVSD is, or an atrioventricular septal defect, which is, we call it [an] AVSD … There’s other names for it … but that’s the one you’ll hear most commonly” (C1, F19). Often the introduction of the term was accompanied by a statement to help parents learn the terms to communicate with their care team in the future; for example, “Whether we use the term double outlet right ventricle or not, his heart will work … in the same way. … I just want you to hear that term. It’s more than [an] anatomy term. It is important for the surgeon after birth in the way that he approaches things (C5, F12)”.

While medical language was consistently used to introduce the diagnosis, plain language was used as a substitute for medical language after the introduction. For example, one clinician introduced a diagnosis by explaining, “This wall did not grow all the way up and there is a hole here. And that hole is called a ventricular septal defect. Now, that’s just Latin for a hole between chambers” (C1, F8). The clinician went on to explain the next steps by exclusively describing the ventricular septal defect as a “hole” rather than using the medical term: “There are different kinds of holes, okay? I don’t know where your sister’s was, but a hole between the pumps is the most common difference in our population” (C1, F8).

While medical language was seemingly introduced to familiarize families, the term used most frequently to refer to CHD throughout fetal cardiology consultations was the plain-language term “heart difference”. One mother reflected on the importance of this word choice during the visit:

Mother: Okay. Can I just tell you how good it feels that you’re calling it a ‘difference’ and not a ‘defect’ because I had that issue with the twins. Doctors sometimes don’t choose their words very well and it makes you feel bad. So, I love that it’s called a ‘difference’.

Clinician: That’s what we call them here, but it’s good to know that that’s a better word for you to hear as well because we’re all different. Everybody’s different, and so just because your heart is formed differently doesn’t mean you’re defective. (C5, F4)

### 3.3. Use of Person-Centered Language

Person-centered language was used most frequently towards the end of consultations (Figure 2). However, some consultations included a person-centered statement at the beginning of the consultation, primarily to ask about the name of the baby or to express empathy; for example, “You’ve got a lot on your plate” (C4, F4). Person-centered language was consistently used when clinicians discussed the implications of anatomic findings towards the end of the consultation, emphasizing the baby as a whole person. As one clinician stated, “Your little guy’s story is his story. There’s nobody else like your son. There are other children with Ebstein anomaly, but your son is your son” (C5, F5).

Person-first language was used by all clinicians. For example, one clinician said, “Typically, people with Tetralogy of Fallot lead full lives but they do have extra medical needs that will require checkups” (C2, F11). There were a few intermittent examples of clinicians also using disease-centered language, most commonly in the setting of telling a story about other patients, especially those with hypoplastic left heart syndrome or Tetralogy of Fallot. For example, “I have other single ventricles who have played baseball and volleyball on high school teams” (C1, F13) or “our Tetralogy of Fallot’s aren’t across the board on any specific medicine” (C2, F11).

### 3.4. Use of Stories as a Person-Centered Language Strategy

Stories were a person-centered language strategy used by three of the five clinicians to communicate about the baby as a whole person. This technique involved telling stories that focused on other patients with CHD as whole people with lives and characteristics beyond their diagnosis. As an example, when discussing a surgical intervention, one clinician shared, “That [surgery] actually is amazingly well-tolerated by children. One child I saw this week who is six who has really no valve function there, we just patched across, scored 18 goals in soccer this year … And he’s the star of the team! So, it turns out that you don’t need to have a valve here when you’re a child” (C1, F8).

Stories also functioned to depict the baby growing up. One clinician explained, “Overall, babies with transposition, you would not be able to pick out which one had this when they’re in kindergarten” (C2, F2). Another said, “I had a patient who was a dancer and continued to dance in college. And she said, ‘It’s really easy. I can dance for hours because you write your own steps. Fast bit, pose, slow, little rest, now I can do it again’” (C1, F1). A third clinician discussed growing up into adulthood:

Clinician: Do you guys watch winter Olympics at all?

Mother: Oh, we have, yeah.

Clinician: Do you know who Shaun White is, the snowboarder?

Mother: Oh, yeah.

Clinician: He has Tetralogy of Fallot.

Mother: I did not know that.

Clinician: So there you go … And they didn’t know, exactly, before he was born … But look at how well Shaun White, how athletic he is. (C3, F11)

## 4. Discussion

This study demonstrates that clinicians use four main discourse styles during initial fetal cardiology consultations: small talk, medical, plain, and person-centered language. Most clinicians used a similar order of discourse styles: small talk at the beginning and end of the consultation, plain language followed by frequent switching between medical and plain throughout the bulk of the interaction, and person-centered language interspersed more toward the end of the visit. The most frequently used discourse styles included plain and a combination of plain and medical. Medical language was used to sensitize families to what they would hear from other clinicians and was typically defined using plain language. When clinicians used person-centered language, they often used it to emphasize the baby as a whole person rather than focusing on the heart difference.

Prior studies have examined how plain and medical language are used in clinical settings. In many other medical specialties, clinicians primarily use medical language [7,16]; in contrast, fetal cardiology clinicians in our study used plain language most frequently. In general, patients do not easily understand medical language, clinicians overestimate patients’ ability to comprehend medical terms, and undefined jargon can lead to confusion and distress [17,18]. Plain language has been identified as a meaningful approach to addressing low health literacy [19], as its use may improve patient comprehension and sense of control in healthcare settings [20,21]. Patients prefer it when clinicians take time to explain the meaning and relevance of medical terms [21], which was done frequently by the clinicians in our study. Introducing medical terms may serve to familiarize parents with them and help them learn the terms to communicate with their care team in the future. While using both medical terms and plain language is important, the potential effects of using these discourse styles during a fetal cardiology visit are not well understood.

The medical community as a whole has moved towards person-first, person-centered, and plain language [6,22]; however, favored language ultimately is determined by personal preference, and communities often differ on their preferences [23,24,25,26,27]. Person-first language is only one component of person-centered language; word choice, personalized counseling, and descriptions of the patient beyond their diagnosis are other components [28]. Prior studies of prenatal medical visits have found that parents express a strong preference for word choice, and words do affect their perceptions [29]. One participant in our study commented directly to the clinician, “Doctors sometimes don’t choose their words very well and it makes you feel bad” (C5, F4). In our study, other person-centered language strategies employed by clinicians included empathetic statements and stories about other patients to recognize families in the context of their lives, including, but not limited to, this diagnosis [30,31]. Clinicians mostly used these person-centered strategies in the second half of the visit; the effects of using this discourse style at a particular time during the visit are not known.

The ideal approach to using certain discourse styles and the order of their use are unclear. Balancing anatomically correct terms with terms that are easy to comprehend and process is challenging. Within the field of pediatric cardiology, parents of children with CHD have requested more information be provided during prenatal counseling visits, including detailed anatomical descriptions. At the same time, parents rank clear and easily understandable communication as one of the most important aspects of a prenatal counseling visit [32,33,34,35,36]. These seemingly competing desires are challenging to integrate into a single communication approach and often lead to conflicting feedback. Trying to meet all the competing needs may leave parents feeling like passive receivers of information [37], which can result in a negative experience of the counseling visit [1,35,36]. Future research should evaluate the effects of various approaches to balancing and sequencing discourse styles in this setting.

### 4.1. Implications

In this study, we described the discourse styles and order of those styles used in initial fetal cardiology consultations. Our findings raise important clinical, research, and educational considerations that could have implications not only for fetal cardiology clinicians but also for other clinicians who deliver prenatal diagnoses.

One potential clinical implication relates to balancing the introduction of medical language and the use of plain language across multiple visits. Clinicians may want to re-evaluate the timing of the topics covered with families prenatally after a CHD diagnosis. Parents’ ability to understand the CHD diagnosis and explain the diagnosis to others is an important goal of prenatal counseling [38]. The question is whether those goals must be accomplished during the first fetal cardiology visit. One challenge is that multiple variables affect communication during this initial visit. A fetal cardiologist is tasked with providing enough information so that parents understand the CHD diagnosis and what that diagnosis means for their family to allow them to make an informed decision about continuing the pregnancy, proceed with genetic testing, and consider which interventions would be appropriate for their family and their baby. To be informed, a family needs to understand their baby’s cardiac anatomy and physiology along with the potential interventions and related complications; however, parents are often experiencing shock, feeling overwhelmed, and struggling to process information at the time of receiving the new diagnosis [39,40]. Future research should address how to tailor counseling sessions to the differing needs of families, potentially providing general information and an outline of future topics to be covered, with the plan to provide parents with more detailed information subsequently [39], rather than trying to cover all the topics recommended to be discussed prenatally [41] in detail during the initial visit. Fetal cardiologists may benefit from identifying as a group the critical elements for the initial consultation and topics that can be covered in subsequent visits.

Additionally, future research will need to assess the nuances of parent and clinician preferences on the balance of medical, plain, and person-centered language use and the potential effects of discourse style on parent and family outcomes. Future investigation should also focus on how to screen for information preferences (e.g., would you prefer to have information in handouts, informational videos, or another modality?) and communication preferences (e.g., would you prefer more details or the big picture?) and then tailor communication to these preferences; we would then need to study whether such interventions improve outcomes. Whether such screening is acceptable, feasible, and effective in this context is an empirical question. Future work should also investigate the use of discourse styles in research publications, as phrasing used in the literature may influence phrasing used in clinical practice; future interventions may address the discourse used in both settings.

From an educational perspective, understanding the conversation structure, specifically the order of discourse styles typically used, will help clinician-educators teach trainees how to approach communication in the fetal cardiology clinic setting. While trainees may naturally progress through similar discourse styles (i.e., from small talk to a combination of plain and medical language with person-centered language interspersed), understanding the structure used in current fetal cardiology clinical practice would facilitate decisions about the language adopted. Future research will need to assess the effects of such clinician-focused education on communication and family outcomes.

### 4.2. Limitations

While prior studies have assessed parental perceptions and clinician-reported practices when counseling about a prenatal CHD diagnosis [1,34,42], this study is one of the first, to our knowledge, to examine the language used in fetal cardiology consultations by analyzing audio-recordings of those encounters [43]. As clinician discourse styles may vary based on prior experiences, training, geographical region, and sociocultural factors, the generalizability of our study findings may be limited as the data analyzed was from one academic institution and limited to five fetal cardiology clinicians. Additionally, the analysis focused on the physician discourse styles and not the interactive discourse between families and clinicians. While it is possible that parents may have influenced discourse styles used by clinicians, analysis of interactive discourse was limited given the imbalance of dialogue, with only 11% of words spoken by parents on average. Finally, the transcriptions analyzed only included those from initial fetal cardiology visits; language use during subsequent prenatal visits may differ.

## 5. Conclusions

In this study, fetal cardiology clinicians from one tertiary care facility used four main discourse styles—small talk, plain, medical, and person-centered—and employed these in a similar order across initial fetal cardiology encounters. Clinicians frequently incorporate plain language into their consultations, using it to define and describe medical language. Clinicians also used person-centered language to emphasize the baby as a whole person beyond their heart differences. Improved understanding of the discourse styles used and order of their use in typical fetal cardiology care highlights further research needed into the ideal timing of, preferences around, and means of educating about these discourse styles. Additionally, future studies should work to understand the effects of clinician discourse styles on family outcomes, including parents’ decision-making, psychological adjustment, and quality of life.

## Figures and Tables

**Table 1:** Participant and consultation characteristics.

	Characteristic	*n*	%
Clinicians (*n* = 5)	Institutions trained at for residency and fellowship, *n*	5	
	Time practicing at current institution median (IQR), years	10 (2)	
Initial Fetal Cardiology Consultations (*n* = 19)	Duration of counselling, median (IQR), minutes	37 (25)	
	Partner(s) present	14	74
	Other children present	3	16
	Language interpreter present	1	5
Pregnant Persons (*n* = 19)	Gestation at first visit, median (IQR), weeks	26 (7)	
	Native language		
	English	15	79
	Spanish	1	5
	Arabic	1	5
	Kurdish	1	5
	Unspecified African Dialect	1	5
	Race/Ethnicity		
	White (European)	14	74
	White (Middle Eastern)	2	11
	Black/African American	2	11
	Hispanic/Latina	1	5
	Family history of CHD	5	26
	Self-identify as a “mother”	19	100
CHD Diagnoses (*n* = 19)	Lower risk of mortality ^1^		
	Coarctation of the Aorta	2	11
	Tetralogy of Fallot	2	11
	Transposition of the Great Arteries (simple)	1	5
	Pulmonary Stenosis	2	11
	Atrioventricular Septal Defect	1	5
	Vessel aneurysm	2	11
	Higher risk of mortality ^1^		
	Ebstein Anomaly (severe)	1	5
	Hypoplastic Left Heart Syndrome	2	11
	Hypoplastic Right Heart Syndrome	2	11
	Transposition of the Great Arteries (complex)	1	5
	Pulmonary Atresia	1	5
	Double outlet right ventricle and other anomalies	1	5
	Autoimmune complete heart block	1	5

^1^ Lower risk of mortality defined as score of ≤5; higher risk of mortality defined as score of >5 on the suggested CHD prognosis scale [14]. Abbreviations: Congenital heart disease—CHD; Interquartile Range—IQR.

**Table 2:** Discourse styles used by clinicians during initial fetal cardiology consultations for congenital heart disease.

Discourse Style	Definition	Use	Examples
Medical Language	Medical terminology or jargon	Used to describe anatomy, procedures, and medications. Also used to familiarize families with the terms	“She might actually be what we call **“balanced”** because of the difference, which is the **pulmonary stenosis**”. (Clinician 4 to Family 4) “This is one of the **heart lesions** that we do see **intrauterine fetal demise** in”. (Clinician 2 to Family 2)
Plain Language	Clear, straight-forward communication using everyday words [6]	Used to define and explain the meaning of medical language	“Once baby’s born, it’s not uncommon that people would hear a **murmur from it, which is just a sound that you hear from differences in how blood flow can move**, especially if you have something with one of the valves that’s different”. (Clinician 4 to Family 4) “We call this … there’s many names and I’m going to put a bunch of terms on here, **hypoplastic, right-heart syndrome, which just means small right heart**”. (Clinician 1 to Family 13) “Instead of being this nice small vessel, some people get **big baggy blood vessels** there. It is called a ductal aneurysm. Okay? And aneurysm sounds like a terrible word, but **what it really means is a big baggy thing**”. (Clinician 1 to Family 17)
		Used as a substitute for medical language	“And though one blood vessel itself is small, so we would say **hypoplasia or smallness** of the pulmonary artery … So the tricuspid valve is small, the pump is **small** and the way out is **small and actually, sealed over**”. (Clinician 1 to Family 13) “We agree with the diagnosis of **the hole in the heart**”. (Clinician 5 to Family 4)
Person-Centered Language	Emphasized the person prior to the disease (i.e., person-first language), or acknowledged the person’s experience as opposed to talking about them objectively	Used to emphasize the baby as a whole person	“We’re all different. Everybody’s different, and **so just because your heart is formed differently doesn’t mean you’re defective**”. (Clinician 5 to Family 4) “His pump is different but I know a lot of very neat people who are like him in our world”. (Clinician 1 to Family 13) “We’re all a little different on the outside. We can be a little different on the inside” (Clinician 1 to Family 17)
		Use of person-first language	“Typically, **people with Tetralogy of Fallot** lead full lives but they do have extra medical needs that will require checkups”. (Clinician 2 to Family 11)
		Use of stories	“But real stories from patients I’ve taken care of, one young lady who was a gymnast and she said, ‘I can vault, but I can’t do floor exercise. That’s too long.’” (Clinician 1 to Family 13)
Small Talk	Polite conversation about non-sensitive matters [15]	Used to establish rapport with the family	Clinician: I’ve lived in [redact] for [redact] years. You would think I would know where everything is and I still don’t. So I was just trying to get my head around where you lived. So between here and your house is how far time-wise, when you get in your car? Mother: Hour and a half. Clinician: Still good amount. Mother: Without traffic. (Clinician 1 to Family 1)

## Data Availability

De-identified data that support the findings of this study are available upon request from the corresponding authors; data-use agreements will need to be completed and approved to share data. The data are not publicly available due to privacy and ethical restrictions.

## Supplementary Materials

The following supporting information can be downloaded at: https://www.mdpi.com/article/10.3390/jcdd10090394/s1, Figure S1: Language used in fetal cardiology consultations, coded from beginning of the consultation to the end with each segment categorized as medical, plain, patient-centered language or small talk.
