# Supplementary material for: “I Just Want You to Hear That Term”: Characterizing Language Used in Fetal Cardiology Consultations
Source: J Cardiovasc Dev Dis. 2023 Sep 13;10(9):394. doi: 10.3390/jcdd10090394 (PMC10531623; doi:10.3390/jcdd10090394)
Supplement: Supplementary file 1 [file jcdd-10-00394-s001.zip › jcdd-2577872-supplementary.pdf]

Supplementary Materials

Figure S1. (**a**,**b**) Language used in fetal cardiology consultations, coded from beginning of the consultation to the end with each segment categorized as medical, plain, patient-centered language or small talk.

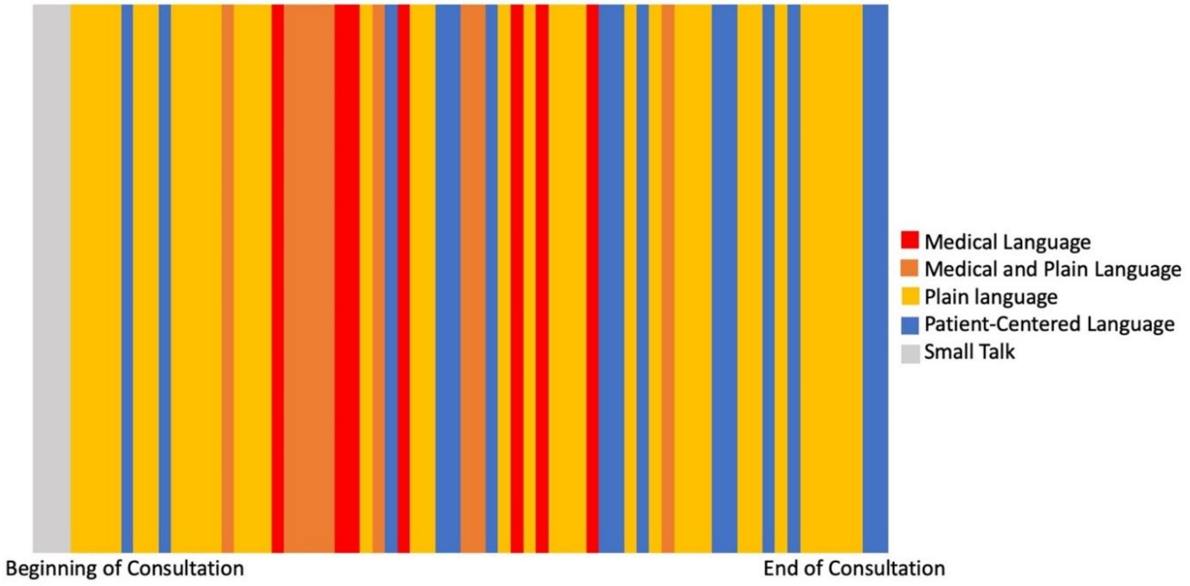

(a)

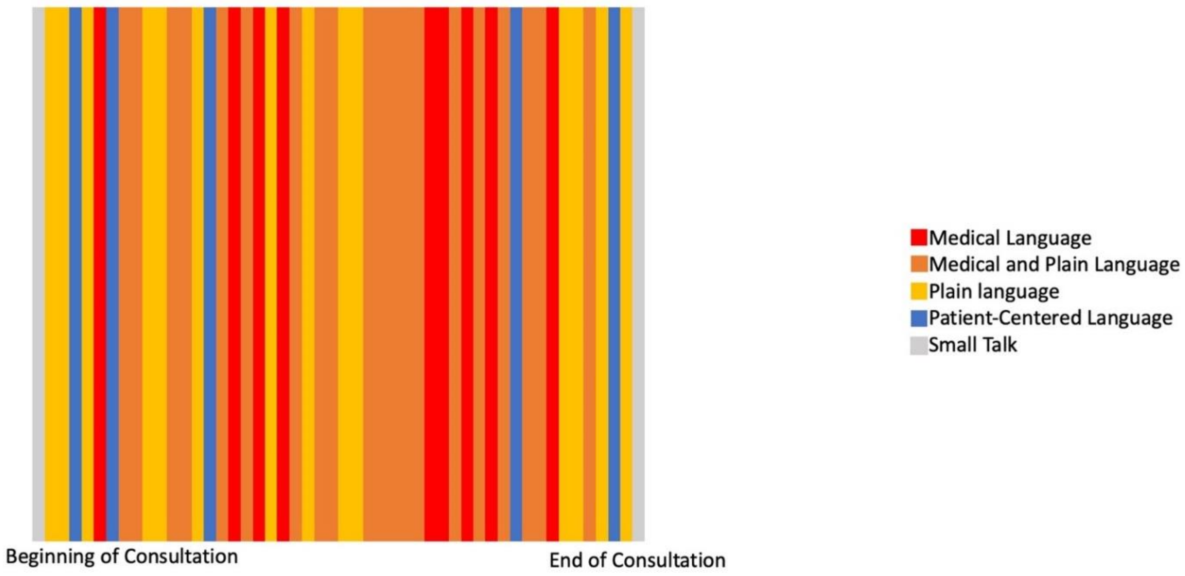

(b)
